# A New Tissue Resonator Indenter Device and Reliability Study

**DOI:** 10.3390/s110101212

**Published:** 2011-01-20

**Authors:** Ming Jia, Jean W. Zu, Alireza Hariri

**Affiliations:** Department of Mechanical & Industrial Engineering, University of Toronto/5 King’s College Road, Toronto, ON M5S 3G8, Canada; E-Mails:zu@mie.utoronto.ca (J.W.Z.); hariri@mie.utoronto.ca (A.H.)

**Keywords:** tissue mechanical properties, TRID, reliability, indenter misalignment

## Abstract

Knowledge of tissue mechanical properties is widely required by medical applications, such as disease diagnostics, surgery operation, simulation, planning, and training. A new portable device, called Tissue Resonator Indenter Device (TRID), has been developed for measurement of regional viscoelastic properties of soft tissues at the Bio-instrument and Biomechanics Lab of the University of Toronto. As a device for soft tissue properties *in-vivo* measurements, the reliability of TRID is crucial. This paper presents TRID’s working principle and the experimental study of TRID’s reliability with respect to inter-reliability, intra-reliability, and the indenter misalignment effect as well.

## 1. Introduction

The measurement of soft tissue mechanical properties is useful in many applications. For centuries, physicians have used palpation as an important diagnostic tool. The efficacy of palpation is based on the fact that many diseases cause changes in tissue mechanical properties. These changes are caused either by exudation of fluids from the vascular system into the extra- and intracellular space or by loss of lymphatic systems, as in the case of cancer. The result is an increase in stiffness or elastic modulus of the tissue. For instance, during many abdominal operations, palpation is used to assess organs, such as the liver, and it is not uncommon for surgeons at the time of laparotomy to palpate tumors that were undetected preoperatively by Computerized Tomography (CT), magnetic resonance (MR), or ultrasound (US). This is because none of these modalities currently provide the type of information elicited by palpation. Quantitative local measurements of mechanical properties of tissues have the potential to provide a method for more accurate tissue classification and early detection of diseases [1].

For several decades, interest in brain tissue mechanical behavior has been increasing, mostly as a result of the emergence of biomedical engineering fields such as head impact biomechanics and neurosurgery simulation [2]. Computational models of traumatic brain injury (TBI) can play an important role to supplement animal models, human surrogate and patient studies in identifying mechanisms of TBI. Relative influence of brain mass, load magnitude, contact surfaces and protective interventions can be explored relatively easily by modifying the computational simulations [3–5]. However, the accuracy of these simulations is strongly dependent on the assumptions and approximations used to model brain tissue material properties [6]. Surgical simulation and training methods are in high demand for new surgical trainees since the use of human subjects, or cadavers and live animals is unrealistic and unethical. Computer-based surgical simulation will provide surgeons with the opportunity to practice specific procedures repeatedly, without depending on patients arriving in an operating room with relevant conditions. To help the surgeon learn the manual tasks properly, and to make the learning experience as realistic as possible, mathematical models, which can accurately describe mechanical behavior of soft tissue undertaking load, are needed. For all of these applications described above, detailed knowledge of the mechanical properties of living tissue is essential.

The existing devices for measuring tissue mechanical properties can be grouped in three main categories: devices relying on imaging techniques; devices using indentation methods; and devices based on vibration principles. There are many examples from the first category, such as Magnetic Resonance Elastography (MRE) [7], Ultrasound (US) tissue-type imaging (TTI) [8,9], and Harmonic Motion Imaging (HMI) [10]. The devices in the first category are really helpful for extracting tissue’s elastic properties. However, they are expensive, bulky, non-portable, and with large error due to the big effect of ambient noise on test results, and/or problems of inferring elastic properties from acquired images, such as fidelity of images, speed of acquiring images, or images being 2D while the strain/stress field is 3D. The basic working principle of devices in the second category is that the force applied to the tissue through an indenter and the indenter’s motion is measured. A device called TeMPeST 1-D, developed by Ottensmeyer [11], uses a cylindrical flat indenter to poke soft tissues with various types of dynamic motions, such as harmonic or chirp, and measures the indentation force and tissue displacement at the indenter tip. By analysing the magnitude and phase of the force over displacement transfer function, this device can provide information about both elastic and viscous part of the tissue behavior. The drawbacks associated with these methods are: they rely on direct measurement of tissue motion which produces reliability issues; breathing and heart beat significantly affect the measured force responses of the organs; and measuring sessions are long with lots of data points required at each frequency due to the reliability problems. All of these issues make these methods less desirable for *in-vivo* measurements. For the third category, some existing devices [12] can only measure tissue elastic properties but not the viscous properties of them. Some others [13] can measure both of them but with too high natural frequencies which are not applicable to most clinical and medical applications with low-frequency range.

**Figure 1.**
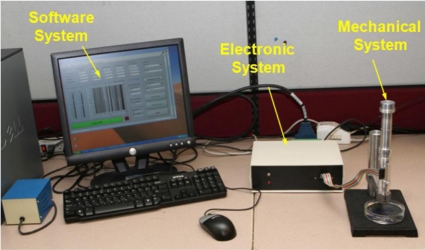
Overall view of the experiments using TRID.

**Figure 2.**
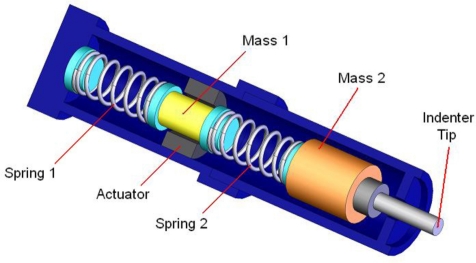
Schematic diagram of the mechanical part of TRID.

**Figure 3.**
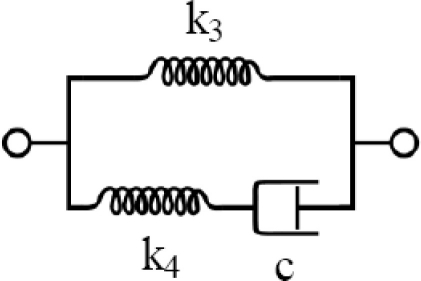
Kelvin model used to model viscoelastic materials.

Given the limitations of existing devices, a new portable device, called Tissue Resonator Indenter Device (TRID), has been designed and prototyped at the Bio-instrument and Biomechanics Lab of the University of Toronto for measuring regional viscoelastic properties of soft tissues in the range of 0–100 Hz [14]. The device is an evolved and completely redesigned version of the idea proposed in [15] and [16]. The overall view of the experiments using TRID can be seen in Figure 1. This device has three main parts: the mechanical system, the electronic system, and the software system installed in a computer. The mechanical properties of soft tissues can be determined by exploiting the fact that they both exhibit springiness (*i.e.*, have stiffness) and dissipative character (*i.e.*, have damping). If an external system with known natural frequencies and damping ratios comes into contact with a soft tissue under study, a shift will be observed in its natural frequencies and its damping ratios will increase. This simple idea is the underlying principle based on which TRID works. For this work, the mechanical system of TRID consists of two springs and masses that are connected back to back to produce a two-degrees-of-freedom system with known natural frequencies and damping ratios, which is shown in Figure 2. When a soft tissue, which is assumed as a Kelvin model [17], comes into contact with the indenter tip of the device, its viscoelastic properties will be felt through the shift in the natural frequencies and damping ratios of the device. Figure 3 shows the Kelvin model which is used to model viscoelastic materials. By providing accurate stress relaxation and creep characteristics, this model can be used to model the viscoelastic soft tissue and thereby calculate the creep and stress relaxation modulus. The three parameters of Kelvin model are the static stiffness *k*_3_, dynamic stiffness *k*_4_, and damping *c* of soft tissues. By obtaining the three unknown tissue parameters, the creep and relaxation modulus quantifying the viscoelastic tissue material can be found. The creep and stress relaxation modulus along with the relaxation and retardation times can be extracted via the mechanical components of the Kelvin model. The relaxation modulus is then given by:

(1) $$E(t)=k_{3}+k_{4}e^{-t/\tau_{r}}$$

where *τ_Y_* is the relaxation time:

(2) $$\tau =\tau_{r}\approx c/k_{4}$$

The creep function is given by:

(3) $$J(t)=\frac{1}{k_{3}}+\frac{k_{4}}{k_{3}\left(k_{4}+k_{3}\right)}e^{-t/\tau_{c}}$$

where *τ_C_* is the creep or retardation time:

(4) $$\tau_{c}=\tau_{r}\frac{\left(k_{4}+k_{3}\right)}{k_{3}}$$

With respect to the measurement, the excitation signal, a pseudo random binary signal (PRBS) which provides better crest factor, is produced by the software system. The signal then passes through the electronic system and gets amplified. The amplified signal is then applied to a voice coil actuator inside the mechanical system. This produces motion in the indenter tip of the device to excite the attached tissue with extremely small amplitude. The system responses, including the linear displacement of mass m1, the acceleration of mass m2, and the force felt by the tissue, are picked up by a LVDT sensor, a MEMS accelerometer, and a miniature force sensor inside the mechanical system respectively. The data picked up by the sensors is then sent back to the software system after passing through an anti-aliasing filter and a band pass filter in the electronic system. With these data, the shift in the natural frequencies and damping ratios of the device can be acquired by using LabView.

Apparently, the reliability of TRID is crucial as a potential clinical apparatus. The first issue is that the measurement result variety of a soft tissue sample should be within a reasonable range with respect to TRID measurements with different users. Another issue is that the TRID measurement result of a soft tissue sample with the same user should be repeatable. With respect to the potential applications of TRID for *in-vivo* measurements, the indenter misalignment will occur definitely, which means the indenter of TRID is not perpendicular to the surface of soft tissues under study. Thus the experimental study of TRID’s reliability with respect to inter-reliability, intra-reliability, and the indenter misalignment effect is performed.

## 2. Theoretical Fundament of Measurement

**Figure 4.**
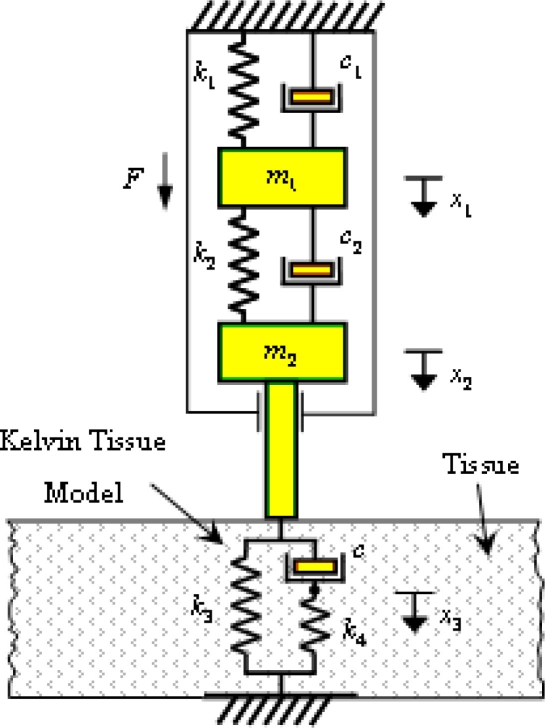
Schematic view of TRID mechanical system contacted with Kelvin tissue model.

Before describing the reliability investigation of TRID, it is necessary to introduce the theoretical fundament of TRID’s measurement, which means how the soft tissues properties are figured out. The schematic view of TRID mechanical system contacted with Kelvin tissue model is shown in Figure 4. The two masses inside TRID’s mechanical system, *m*_1_ and *m*_2_, are connected by two compression springs whose stiffness are *k*_1_ and *k*_2_. Except the two compression springs, there are two viscous dampers *c*_1_ and *c*_2_ between the two masses, which account for the damping effects within the mechanical system. For the whole system shown in Figure 4, the equation of motion can be written in the following matrix form:

(5) $$M\ddot{X}+C\dot{X}+\mathrm{KX}=F$$

where:

(6) $$M=\left[\begin{array}{ccc} 1 & 0 & 0\\ 0 & 1 & 0\\ 0 & 0 & 0 \end{array}\right],\;\;\;C=\left[\begin{array}{ccc} \phi +m\psi & -m\psi & 0\\ -\psi & \psi & 0\\ 0 & -1 & 1 \end{array}\right]$$

and:

(7) $$K=\left[\begin{array}{ccc} \alpha +m\beta & -m\beta & 0\\ -\beta & \beta +\Gamma & \Theta \\ 0 & 0 & \eta \end{array}\right],\;\;F=\left\{\begin{array}{c} F/m_{1}\\ 0\\ 0 \end{array}\right\},\;X=\left\{\begin{array}{c} x_{1}\\ x_{2}\\ x_{3} \end{array}\right\}$$

The parameters are defined as follows:

(8) $$\begin{array}{l} \alpha =\frac{k_{1}}{m_{1}},\;\;\beta =\frac{k_{2}}{m_{2}},\;\;m=\frac{m_{2}}{m_{1}},\;\;\phi =\frac{c_{1}}{m_{1}},\;\;\\ \psi =\frac{c_{2}}{m_{2}},\;\;\Gamma =\frac{k_{3}}{m_{2}},\;\;\eta =\frac{k_{4}}{c},\;\;\Theta =\frac{k_{4}}{m_{2}} \end{array}$$

TRID has two main modes of operation. In the first mode, which is called calibration mode, the device operates without any tissue attached to it. The purpose of this mode is to identify accurately the values of parameters of TRID, including *k*_1_, *k*_2_, *m*_1_, *m*_2_, *c*_1_, and *c*_2_. The typical obtained values of k_1_, k_2_, c_1_, & c_2_ are 3,500 N/m, 800 N/m, 0.5 Ns/m and 0.5 Ns/m. These values change after each calibration with 5% tolerance. In the second mode, which is called tissue test mode, the TRID is brought into contact with a tissue under study. Based on the values of system parameters being obtained in calibration mode, a method is developed for finding the unknown tissue parameters using input-output data of TRID system in time-domain. The TRID system is modeled using physical insight in a grey-box state-space form [18,19]. Since the input and outputs of the system are contaminated with noise, a Kalman filter predictor is constructed for predicting the future values of the outputs. The prediction errors are then computed and minimized using prediction error method, resulting in the unknown parameters of the TRID system and the tissue under study. For physically parameterized systems, it is natural to work with the equation in state-space form. Otherwise, for example in transfer function form, the parameters will combine and produce nonlinear coefficients, rendering this approach useless. The continuous-time stochastic state-space equations of a dynamic system is usually presented as follows:

(9) $$\begin{array}{l} \dot{x}(t)=\mathrm{Fx}(t)+G_{c}u(t)+w(t)\\ y(t)=H_{c}x(t)+v(t) \end{array}$$

where *x*_*n*×1_ is the state vector, *u*_*p*×1_ is the input vector, *y*_*q*×1_ is the output vector, *F*_*n*×*n*_ is the state matrix, *G*_*c n*×*p*_ is the input-to-state matrix, *H*_*c q*×*n*_ is the state-to-output matrix, and *w*_*n*×1_ and *v*_*q*×1_ are process disturbance and output noise, respectively, with the following covariance and cross-covariance matrices:

(10) $$\begin{array}{l} E\left[w(t)w{\left(t+\tau \right)}^{T}\right]=R_{w}(t,\tau )\\ E\left[v(t)v{\left(t+\tau \right)}^{T}\right]=R_{v}(t,\tau )\\ E\left[w(t)v{\left(t+\tau \right)}^{T}\right]=R_{\mathrm{wv}}(t,\tau ) \end{array}$$

where:

(11) $$E\left[a(t)b{\left(t+\tau \right)}^{T}\right]=\int_{t}a(t)b(t+\tau )f_{\mathrm{ab}}(t,\tau )d_{t}$$

and where *f*_ab_ is the joint probability distribution of *a*(*t*) and *b*(*t* + *τ*).

Using Equations (6) and (7), the system matrices in calibration mode will take the following form:

(12) $$\begin{array}{l} F=\left[\begin{array}{cccc} 0 & 1 & 0 & 0\\ -(\alpha +m\beta ) & -(\phi +m\psi ) & m\beta & m\psi \\ 0 & 0 & 0 & 1\\ \beta & \psi & -\beta & -\psi \end{array}\right]\\ G_{c}={\left[\begin{array}{cccc} 0 & c_{1} & 0 & 0 \end{array}\right]}^{T}\\ H_{c}=\left[\begin{array}{cccc} 1 & 0 & 0 & 0\\ 0 & 0 & 1 & 0 \end{array}\right]\\ x={\left[\begin{array}{cccc} x_{1} & {\dot{x}}_{1} & x_{2} & {\dot{x}}_{2} \end{array}\right]}^{T}\\ \theta ={\left[\begin{array}{cccccc} \alpha & \beta & m & \varphi & \psi & c_{1} \end{array}\right]}^{T} \end{array}$$

In the tissue test mode, the number of states increases by one, and we get:

(13) $$\begin{array}{l} F=\left[\begin{array}{ccccc} 0 & 1 & 0 & 0 & 0\\ -(\alpha +m\beta ) & -(\phi +m\psi ) & m\beta & m\psi & 0\\ 0 & 0 & 0 & 1 & 0\\ \beta & \psi & -\beta -\Gamma & -\psi & -\Theta \\ 0 & 0 & 0 & 1 & -\eta \end{array}\right]\\ G_{c}={\left[\begin{array}{ccccc} 0 & c_{2} & 0 & 0 & 0 \end{array}\right]}^{T}\\ H_{c}=\left[\begin{array}{ccccc} 1 & 0 & 0 & 0 & 0\\ 0 & 0 & 1 & 0 & 0 \end{array}\right]\\ x={\left[\begin{array}{ccccc} x_{1} & {\dot{x}}_{1} & x_{2} & {\dot{x}}_{2} & x_{3} \end{array}\right]}^{T}\\ \theta ={\left[\begin{array}{cccc} \Gamma & \Theta & \eta & c_{2} \end{array}\right]}^{T} \end{array}$$

In the above equations, *θ* is the vector of parameters that have to be identified in each case, and *c*_1_ and *c*_2_ are included for the DC gain in each case. The model structure, considering only the deterministic part, is defined as follows:

(14) $$M(\theta )=\left\{(F(\theta ),G_{c}(\theta ),H_{c}(\theta ))|\theta \in D_{\theta }\right\}$$

where *D_θ_* is the domain over which *θ* is defined. The domain *D_θ_* is chosen such that the model is stable. It is noted that in the above equations, the actuator dynamic is neglected. According to the manufacturer the voice coil actuator used here has a first-order dynamics with time constant of *τ* = 1.86 ms:

(15) $$G_{a}(s)=\frac{1}{0.00186s+1}$$

This accounts for a pole at *s* = 537 Hz. Two methods can be employed for dealing with this pole. The first order transfer function (Equation (14)) is an approximation of a pure time delay of *e*^−0.00186^*s*. Therefore, the system can be sampled with a sampling interval of *τ* /*b* (*b* is an integer greater than 1), and then the calibration mode (Equation (11)) and tissue test mode (Equation (12)) (and thereby ignoring the actuator dynamic) can be used with considering *b* number of delays between input and outputs.

A more accurate approach is to still sample the system with a sampling interval *τ* /*b* (*b* is now a real number greater than 2), but use the following input for performing the identification:

(16) $$u_{a}(s)=u(s)G_{a}(s)$$

where *u* is the original output used for exciting the system. The calibration mode (Equation (11)) and tissue test mode (Equation (12)) used with this new input will be equivalent to the system with the actuator dynamic and original input. It can also be interpreted as passing the input and outputs of the calibration mode (Equation (11)) and tissue test mode (Equation (12)) through the same prefilter *Ga*(*s*). With respect to TRID, we employ the second approach as it is more accurate and imposes less restriction on the sampling interval.

The prediction error method [20], which contains maximum likelihood (ML) method [21,22] as a special case, is a natural choice in many applications for finding the parameter estimates. As the name implies, it minimizes the prediction errors (more precisely, their norms), and provides unbiased estimates when the true system is contained in *D_θ_* [23]. In this approach, a predictor model of the system is constructed first. Then, a specific norm of the prediction is minimized using numerical algorithms.

Since the input-output data is obtained in discrete time instants, it is easiest to work with system equations in discrete-time domain. In this case, the system is usually presented using stochastic linear difference equations [24]:

(17) $$\begin{array}{l} x_{k+1}=A(\theta )x_{k}+B(\theta )u_{k}+w_{k}\\ y_{k}=C(\theta )x_{k}+v_{k} \end{array}$$

where (.)*k* = (.)(*kT*), *kT* ≤ *t* < (*k* + 1)*T*, and *T* is the sampling time. The system matrices are obtained using matrices in Equation (9):

(18) $$\begin{array}{l} A(\theta )=e^{F(\theta )T}\\ B(\theta )=\int_{t=0}^{T}e^{F(\theta )t}G_{c}(\theta )\mathrm{dt}\\ C(\theta )=H(\theta ) \end{array}$$

One can derive the disturbance and noise sequences *w_k_* and *v_k_* from their continuous-time counterpart in Equation (9), if s\he has specific insight about their properties in time domain. Otherwise, they are assumed to be sequences of independent identically distributed (i.i.d) random variables with zero means and covariance matrices as follows:

(19) $$\begin{array}{l} E\left[w_{i}w_{j}^{T}\right]=R_{1}(\theta )\delta_{\mathrm{ij}}\\ E\left[v_{i}v_{j}^{T}\right]=R_{2}(\theta )\delta_{\mathrm{ij}}\\ E\left[w_{i}v_{j}^{T}\right]=R_{12}(\theta )\delta_{\mathrm{ij}} \end{array}$$

To predict future values, assuming *w_k_* and *v_k_* are Gaussian processes, we use the stationary Kalman filter [24,25] to recursively find the predicted values of states and output at each time step from the knowledge of previous time instants:

(20) $$\begin{array}{l} x_{k+1|k}(\theta )=A(\theta )x_{k|k-1}(\theta )+B(\theta )u_{k}+K_{k}(\theta )\times \left[y_{k}-C(\theta ){\hat{x}}_{k|k-1}(\theta )\right]\\ y_{k|k-1}(\theta )=C(\theta )x_{k|k-1}(\theta ) \end{array}$$

In the above equation, the usual two-part Kalman filter consisting of measurement-update and time-update is combined into one equation. Here, *ξ̂*_*k|k*−1_ is the estimated value of dummy variable *ξ* at time instant *k* (*ξ* can be x or y), given all the input-output date up to and including the time instant *k*–1. In Equation (19) *K_k_* is the Kalman gain found using the noise second order properties given in Equation (14):

(21) $$\Sigma_{k|k-1}(\theta )=E{\text{cos}}^{-1}\theta \left[\left[x_{k}-x_{k|k-1}(\theta )\right]\;{\left[x_{k}-x_{k|k-1}(\theta )\right]}^{T}\right]$$

where ∑_*k|k*−1_ is covariance of state estimate errors:

(22) $$K_{k}(\theta )=\left[A(\theta )\Sigma_{k|k-1}(\theta )C^{T}(\theta )+R_{12}(\theta )\right]\times {\left[C(\theta )\Sigma_{k|k-1}(\theta )C^{T}(\theta )+R_{2}(\theta )\right]}^{-1}$$

and is obtained recursively by solving the discrete-time Riccati equation:

(23) $$\Sigma_{k+1|k}=A\Sigma_{k|k-1}A^{T}+R_{1}-K_{k}\left[R_{2}+C\Sigma_{k|k-1}C^{T}\right]K_{k}^{T}$$

where the argument *θ* is dropped for brevity. For convenience, the conditional term |*k* is dropped from the parameters. Therefore, *x*_*k*+1_ is the short form for *x*_*k*+1|*k*_.

The prediction errors are found as:

(24) $$e_{k}(\theta )=y_{k}-y_{k}(\theta )=y_{k}-C(\theta )x_{k}(\theta )$$

It is also called innovations as it is a part of output that cannot be estimated from the past data. In innovations form, Equation (19) will take the following form:

(25) $$\begin{array}{l} x_{k+1|k}(\theta )=A(\theta )x_{k|k-1}(\theta )+B(\theta )u_{k}+K_{k}(\theta )e_{k}(\theta )\\ y_{k}=C(\theta ){\hat{x}}_{k|k-1}(\theta )+e_{k}(\theta ) \end{array}$$

Now assuming an input-output sequence for *N* consecutive observations, *z_N_* = [*u*_1_…*u*_N_ *y*_1_…*y_N_*]*T*, we form the following norm from the prediction error sequence:

(26) $$V_{N}\;(\theta ,z_{N})=\frac{1}{N}\sum_{k=1}^{N}L\left(e_{k}\;(\theta )\right)$$

According to prediction error method, the estimate of parameters is the value that minimizes the above norm [23]:

(27) $${\hat{\theta }}_{N}=\underset{\theta \in D_{\theta }}{\text{arg min}}\;V_{N}\left(\theta ,z_{N}\right)$$

In Equation (25) *L*(.) can be any norm, but it is usually considered to be the L_2_ norm (*i.e.*, *L*(*e_k_*) = 1/2*e_k_*^2^) resulting in the least square (LS) criterion. The L_2_ norm can be also arrived at by employing the ML method and considering the innovations to be Gaussian. Equation (26) must be solved using numerical iterative search methods as no closed form analytical solution can be found for it. Here, the Gauss-Newton method [26] is chosen for this purpose. In general, an iterative search method will start from an initial guess *θ*_0_ and proceeds as follows:

(28) $$\delta \theta =-H^{-1}g$$

According to Newton’s method for minimizing the function *V*, *δθ* is the increment in each step and is obtained as follows:

(29) $$\theta^{i+1}=\theta^{i}+\delta \theta$$

where **g** is the gradient of *V* with respect to θ and **H** is its Hessian. The Gauss-Newton method is obtained by ignoring the second order derivatives in the Hessian. Consequently, the Gauss-Newton method is written as:

(30) $$\theta^{i+1}=\theta^{i}-\frac{1}{N^{2}}{\left(\sum_{k=1}^{N}\frac{\partial e_{k}}{\partial \theta }\frac{{\partial e}_{k}}{\partial \theta }\right)}^{-1}\sum_{k=1}^{N}\frac{{\partial e}_{k}}{\partial \theta }e_{k}$$

## 3. Experimental Study of Indenter Misalignment

The indentation test is an effective and relatively simple way to make biomechanical assessment of the skin and subcutaneous tissues under compression. Indenter misalignment effect is not a problem for those earlier apparatus since their indenters were driven and aligned by some type of mechanical devices. However, this made them too clumsy to be feasible for extensive clinical application. TRID, as a pen-size hand-held device, was developed to overcome some of these shortcomings. Using TRID, *in-vivo* indentation tests can be conducted by hand through pushing the indenter on the surface of soft tissues. This kind of indentation test performed without any attachment device is a more clinically feasible way for the biomechanical assessment of the soft tissue *in vivo*. However, it is difficult to avoid the misalignment of the indenter all together, because the soft tissue layers and the underlying bone usually have curved surface. Therefore indenter misalignment will be caused by the non-perpendicular punching of the indenter or the non-parallel tissue layer. Since indenter misalignment is inevitable for hand-held devices like TRID, its effect on the recorded indentation response should be well studied to make sure whether the experimental results are reliable with different angles between the indenter and the tissue surface.

**Figure 5.**
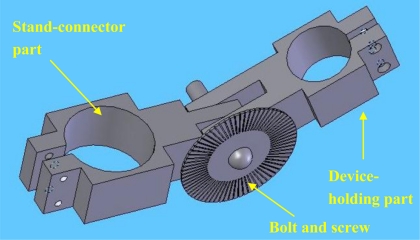
CAD model of designed apparatus part for misalignment experiment.

In order to perform the indenter misalignment experiment, a mechanical device, which can hold the mechanical system of TRID and regulate the angle between the indenter and the surface of soft tissues as well, has been developed (Figure 5). This apparatus consists of two parts: the device-holding part and the stand-connector part. A bolt and a screw, which can allow the two parts to rotate around each other, hold the parts together and can also fix the parts with respect to one another at different angles. A small plate with angular measurements is placed around the bolt and is used to align the angle between the two parts. One silicone gel sample with cylindrical shape was selected as tissue phantom to be measured using TRID. The diameter of the silicone gel sample is 45 mm and the height of it is 30 mm. The silicone gel sample is made of RTV 6166 silicone gel, produced by Momentive Inc. RTV silicone gel can cure to form very soft, gel-like elastomers, which have outstanding stress-relief properties, Mechanical shock/vibration dampening properties, and excellent moisture protection properties. The silicone gel samples have similar properties as those of soft tissues with stable performance within a big range of ambient conditions. RTV silicone gel is made from two liquid portion called A and B. Different proportions of A and B results in silicone gels with different modulus. The A/B proportion of the silicone gel sample used in misalignment experiment is 30/70 and the Elastic Modulus of this sample is 17.5 kPa.

**Figure 6.**
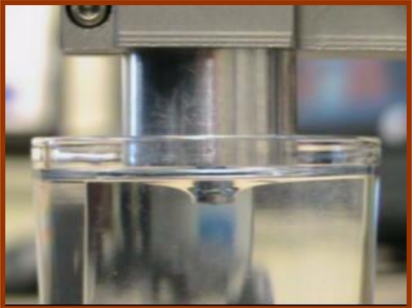
The overall view of the misalignment experiment.

The overall view of the misalignment experiment can be seen in Figure 6. The mechanical system of TRID is held by the apparatus’s device-holding part. The indenter was aligned to be perpendicular to the silicone gel sample’s upper surface initially. After being calibrated, TRID is brought to contact with the silicone gel sample at the center point on the sample’s upper surface with different misalignment angles: 0, 2.5, 5.0, 7.5, 10.0, 12.5 and 15.0 degrees, respectively. The input signal amplitude of 0.5 V is selected, with which TRID can provide more accurate measurements. This has already been proved in our past work [27]. The indentation depth is 1 mm. Misalignment experiments with indentation depth larger than 1 mm can’t be performed yet since there will be a conflict between TRID’s mechanical system and soft tissue surface in the case of large misalignment angle being applied. This problem can be solved in the future by changing the tip shape of TRID’s mechanical system. Three tests were performed on the same point on upper surface of the silicone gel sample for each misalignment angle. Thus, based on the experimental results, how the change of misalignment angles can affect the measurements of soft tissue mechanical properties will be figured out.

**Figure 7.**
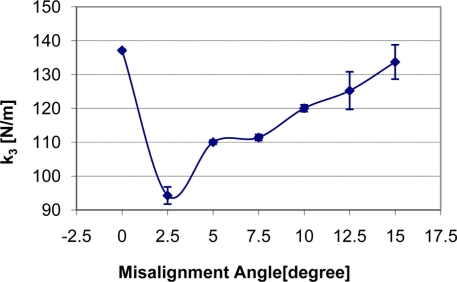
Indenter misalignment effect on static stiffness k_3_.

**Figure 8.**
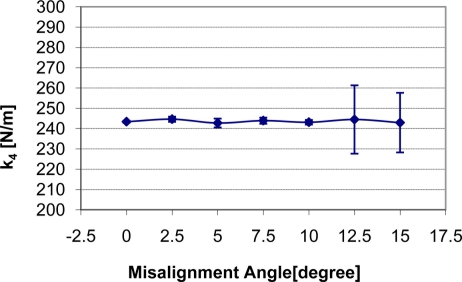
Indenter misalignment effect on dynamic stiffness k_4_.

**Figure 9.**
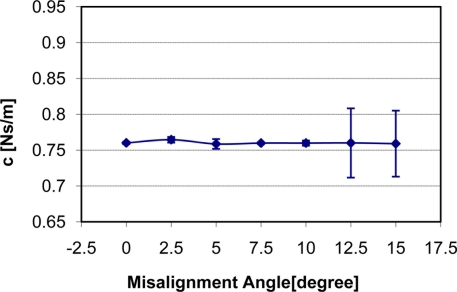
Indenter misalignment effect on damping C.

How the change of misalignment angle affects static stiffness k_3_, dynamic stiffness k_4,_ and damping c of the silicone gel sample with input signal amplitude of 0.5 V is shown in Figures 7–9 respectively. With respect to the misalignment experimental results, there are apparent fluctuations in the values of static stiffness with varying off-perpendicular contact angles. The main cause of this phenomenon is the friction between the indenter of TRID and its bearing. Figure 7 shows that the friction has the biggest effect on experimental results with misalignment angle 2.5 degree. However, the values of the dynamic stiffness and the damping for varying off-perpendicular contact angles ranging from 0 degree to 15 degree have remained approximately constant. This means that the dynamic measurement method of the TRID has advantages for measurement with varying off perpendicular contact angles compared with the static measurement method of other devices. However, this reliability investigation was performed with silicone gel samples which are homogeneous and isotropic. If it were performed with real soft tissues, which are non-homogeneous and/or anisotropic, the results would be different. Therefore, the reliability investigation should be performed with real tissues such as animal organs or muscles in the future before TRID will be developed into a reliable clinical application tool.

## 4. Experimental Study of Inter-Reliability and Intra-Reliability

The purpose of experimental study of TRID’s intra-reliability is to investigate whether its measurements on the same point of the same soft tissue sample are repeatable. While the purpose of experimental study of TRID’s inter-reliability is to investigate how large the difference will be between the measurements with different users of the same soft tissue sample. In this case, another silicone gel sample with cylindrical shape was selected as tissue phantom to be measured using TRID. The diameter of the silicone gel sample is 93 mm and the height of it is 12 mm. The A/B proportion of the silicone gel sample used in misalignment experiment is 50/50 and the Elastic Modulus of this sample is 2.39 kPa. With respect to the inter-reliability experiment, two students of the Bio-instrument and Biomechanics Lab of the University of Toronto were selected as participants. One of the two students, who is called investigator 1, is quite familiar with TRID and has used this device to measure soft tissue mechanical properties for more than 1 year. The other student, who is called investigator 2, has no experience of using TRID before and received a short training about how to use this device to measure soft tissue mechanical properties. Student 1 can be assumed as a professional user of TRID and student 2 can be assumed as a normal consumer of TRID in the future. Thus the inter-reliability of TRID based on the measurements of these two students can be used to investigate whether or not TRID has the possibility to be developed as a commercialized household consumer product. The two students’ measurements have the same experimental procedures and ambient conditions. With respect to each student’s measurements, after being calibrated, TRID is brought to contact with the silicone gel sample at the center point on the sample’s upper surface with different indentation depth: 1 mm, 2 mm, 3 mm, 4 mm, and 5 mm respectively. The input signal amplitude of 0.5 V is selected due to concerning the accuracy of TRID. Three tests were performed on the same point on upper surface of the silicone gel sample for each indentation depth. Therefore, based on the experimental results, the intra-reliability and inter-reliability of TRID will be figured out.

**Figure 10.**
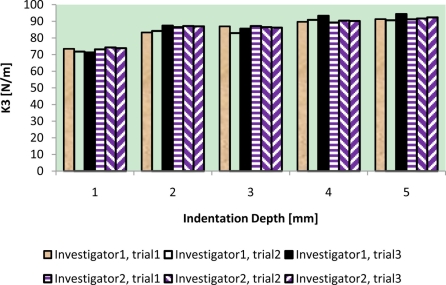
Intra-reliability and inter-reliability experiment of static stiffness k_3_.

**Figure 11.**
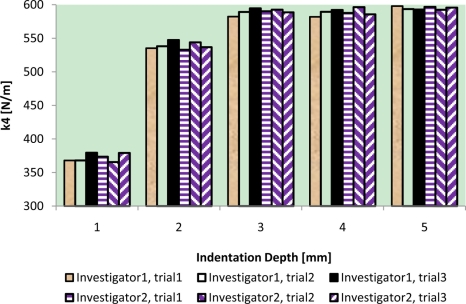
Intra-reliability and inter-reliability experiment of dynamic stiffness k_4_.

**Figure 12.**
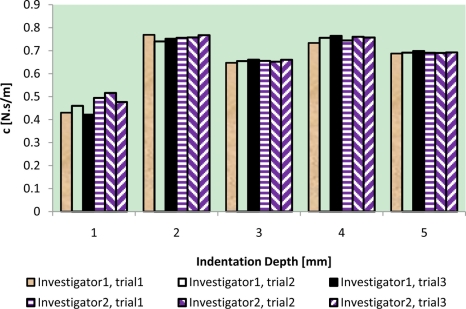
Intra-reliability and inter-reliability experiment of damping C.

The results of intra-reliability and inter-reliability experiment are shown in Figures 10–12.

These figures show how TRID measurements of static stiffness k_3_, dynamic stiffness k_4,_ and damping c of the silicone gel sample change with respect to different trials and investigators. For static stiffness k_3_, intraclass correlation coefficient (ICC) of investigator 1 is 0.95, ICC of investigator 2 is 0.99, and ICC for inter-rater reliability is 0.96. For dynamic stiffness k_4_, ICC of investigator 1 is 0.99, ICC of investigator 2 is 0.99, and ICC for inter-rater reliability is 0.99. For damping c, ICC of investigator 1 is 0.98, ICC of investigator 2 is 0.99, and ICC for inter-rater reliability is 0.97. All these indicate high reproducibility of TRID’s measurements between trials and investigators.

## 5. Conclusions

A new hand-held device TRID has been developed successfully to measure soft tissues mechanical properties. As a potential commercialized household consumer product, TRID needs to be portable, easy to use, and reliable. Among these attributes, reliability of TRID is crucial and should be investigated before TRID being developed as a consumer product in the real world. For this purpose, experimental study was performed to investigate TRID’s reliability with respect to the indenter misalignment and reproducibility of TRID’s measurements between trials and investigators. The experimental results show that indenter misalignment has extremely small effect on TRID’s measurements of soft tissue dynamic properties, which means TRID can be developed as a reliable clinical application for diagnostics of disease. The experimental results also have proven that TRID is reliable between trials and users. This is very important since it means that TRID has the possibility to be developed as a commercialized household consumer product. Another factor that has effect on TRID’s reliability is the contact force between the indenter and soft tissue. The investigation of the effect of the contact force has been described in [27]. However there is still room for the improvement of TRID reliability investigation. In the future, finite element analysis will be performed to investigate the effect of indenter misalignment. Indenter misalignment experiments with indentation depth more than 1 mm can also be performed.
